# Sleep: A Game Changer for Youth Athlete Wellbeing

**DOI:** 10.1177/19417381251350693

**Published:** 2025-07-08

**Authors:** Tanisha Tate, Luana C. Main, Spencer Roberts, Lyndell Bruce

**Affiliations:** † Centre for Sport Research, Institute for Physical Activity and Nutrition, Deakin University, Burwood, Victoria, Australia; ‡ Institute for Physical Activity and Nutrition, School of Exercise and Nutrition Sciences, Deakin University Burwood, Victoria, Australia

**Keywords:** actigraphy, exercise, fatigue, recovery, stress

## Abstract

**Background:**

Youth athletes may have reduced opportunities for sufficient sleep, and often report poor mental health and wellbeing.

**Hypothesis:**

Most youth athletes obtain less than recommended duration and quality of sleep, with associated reduced wellbeing.

**Study Design:**

Prospective observational study.

**Level of Evidence:**

Level 3.

**Methods:**

Sleep was monitored in 98 youth athletes (15.19 ± 2.08 years) for 8 consecutive weeks. Wellbeing was self-reported weekly using the Multicomponent Training Distress Scale (MTDS) and the Short Recovery Stress Scale (SRSS).

**Results:**

Adolescent athletes averaged 7.0 (±1.1) hours sleep per night, but did not meet recommended daily sleep duration (ie, ≥8 hours) on >80% of nights studied. Significant sleep disturbances were detected in 40% of participants. Total sleep time measured via actigraphy was associated negatively with MTDS depression (all values given as [β; CI)] (−0.50; −0.95−0.05), stress (−0.78; −1.23−0.33) and sleep disturbance (−0.62; −0.97−0.27) subscales and overall score (−2.57; −4.27−0.87), and the SRSS stress subscale (−0.82; −1.50−0.15) (*P* < 0.05). Subjective sleep quality measured via sleep diaries was associated positively with all MTDS scores including depression (0.85; 0.50-1.19), sleep disturbance (1.04; 0.72-1.35), and physical symptom (0.60; 0.13-1.08) subscales, and the SRSS stress subscale (1.67; 0.91-2.43), and negatively with SRSS recovery subscale (−1.02; −1.64−0.41). Fatigue ratings before bed and upon awakening were associated positively with all MTDS subscales and the SRSS stress subscale (1.23; 0.74-1.71) (1.26; 0.79-1.74) (*P* < 0.05).

**Conclusion:**

Many adolescent athletes do not meet current sleep recommendations, and inadequate duration and/or quality of sleep in this population is associated with poorer self-reported wellbeing.

**Clinical Relevance:**

Education surrounding sleep in youth athletes should emphasize the importance of adequate sleep, alongside the maintenance of regular sleeping patterns to promote wellbeing and mental health.

Healthy sleep plays an important role in maintaining mental health and wellbeing. Adolescents often do not achieve the recommended minimum of 8 hours of total sleep time and/or 85% sleep efficiency, which is important for optimal health.^2,19,32,41^ Several studies have indicated that sleep is associated with the mental health and wellbeing of adolescents; for example, later bedtimes, longer sleep onset latency, and lower sleep efficiency are linked to poorer wellbeing and negative mood states,^6,39^ and irregular sleep phases are associated with depressive symptoms.^11^

Mental health can be referred to as a “state of wellbeing that enables people to cope with the stressors of life, realize their abilities, learn and work well, and contribute to their community.”^50^ Among the general population, 1 in 7 adolescents are likely to develop a mental health issue, such as depression or anxiety.^51^ In addition, research across a large sample of youth athletes identified that 16.9% were experiencing a mental health issue.^49^ More recent studies have highlighted that the mental health of youth athletes remains an important issue, as global events such as COVID-19 have been shown to negatively affect youth athletes’ mental health.^31^ However, a follow-up study conducted 2 years later revealed improvements in the mental health of youth athletes.^48^ Despite this progress, reported levels of anxiety, depression, and quality of life were still higher than pre-COVID levels.^48^ Therefore, it is extremely important to understand the underlying factors contributing to adolescents’ mental health, including factors that may be unique to certain subgroups, such as youth athletes. Given youth athletes face the unique challenge of managing schooling alongside their sporting commitments, they may be especially at-risk of experiencing poor sleep and wellbeing due to competing interests on their time.

To date, few studies have examined the relationship between sleep and wellbeing in youth athletes, and those that have, have relied mostly on subjective sleep assessments, which are susceptible to bias.^12,16,36-38,46,47^ Self-reported sleep quality and sleep duration had a small but significant effect on subjective measures of fatigue, mood, and perceived recovery across a range of team sports.^36^ Watson and Brickson^46^ found a significant improvement in measures of self-reported wellbeing after a night of increased sleep (duration) and greater sleep quality in youth female soccer players. Kölling et al^25^ used actigraphy to assess sleep and recovery objectively in elite youth rowers during a training camp, and found that reduced sleep time was associated with a decrease in perceived recovery and increase in negative emotional state as measured by the Short Recovery Stress Scale (SRSS).^25^ Interestingly, Steenekamp et al^42^ measured the sleep of youth swimmers and rowers with activity monitors and found no association between sleep and subjective ratings of stress, fatigue, or muscle soreness. Collectively, this literature suggests sleep may be associated with youth/adolescent wellbeing; however, studies to date have either used subjective sleep assessments susceptible to bias (eg, social desirability, recall),^16^ or monitored sleep and wellbeing for relatively short periods that may not be representative of habitual sleep.

Research has shown sex differences for sleep duration and sleep efficiency measures,^20^ as well as sleep quality and sleep onset latency.^45^ Other research has shown no sex differences for bedtime, wake up time, sleep duration, or sleep onset latency.^3,27^ With regards to sex differences in the wellbeing of adolescents, Gomez-Baya et al^14^ indicated that boys showed greater confidence, and lower depressive and anxious symptoms when compared with girls. However, this study did not monitor youth athletes, highlighting the need for longitudinal research using both objective and subjective measures to examine male and female differences in sleep and wellbeing in a youth athletic cohort. Given the sex differences for sleep and wellbeing when investigated separately, sexes may exhibit different associations between sleep and wellbeing based on their unique circumstances. The primary aim of this study was to characterize the sleep and wellbeing of youth athletes from multiple sports longitudinally and examine whether there were relationships between measures of sleep and subjective wellbeing. A secondary aim was to explore whether there were any differences in sleep and wellbeing among youth athletes between the sexes.

## Methods

### Participants

A total of 168 youth athletes volunteered to participate in this study. From this cohort, 98 athletes (50 male and 48 female) were included in the analysis (age, 15.19 ± 2.08 years; height, 173.82 ± 10.35 cm; weight, 64.30 ± 12.03 kg) as they provided >50% of data across the duration of the study (ie, a minimum 28 nights of sleep). Participants main sports included netball (n = 23), swimming (n = 6), cricket (n = 6), basketball (n = 27), Australian rules football (AF) (n = 31), and golf (n = 5). Athletes were excluded if they had any previously diagnosed sleep disorders. They were asked to self-report any sleep disorders during screening, but none were reported. Ethical approval was obtained before the study from Deakin University human ethics committee and the Victorian Department of Education ethics review board. Participants and their parents/guardians were provided with a written and verbal explanation of the study procedures before giving informed consent to participate.

### Data Collection

The wellbeing and sleep of participants was monitored over an 8-week period within a school term. Activity monitors measuring sleep variables were worn nightly, sleep diaries were completed daily, and wellbeing surveys were completed every Monday. Baseline measures of subjective sleep quality, sleep hygiene, and circadian phenotype were recorded at commencement of data collection.

### Baseline Sleep Measures

The Athlete Sleep Behavior Questionnaire (ASBQ) and Pittsburgh Sleep Quality Index (PSQI) were completed at baseline to assess participants’ sleep hygiene and sleep quality, respectively.^9,13^ The ASBQ is an 18-item questionnaire where questions are summed to create a global score ranging between 18 and 90. Scores ≤36 represent “good” sleep behavior, scores between 37 and 41 indicate “moderate” sleep behavior and scores ≥42 signify “poor” sleep behavior. Question 4 is associated with alcohol consumption and was removed from the questionnaire before administration at the request of the ethics review group. The ASBQ has been shown to be a reliable tool and has been used previously in youth athletes.^4,44^ The PSQI is a validated questionnaire and asks participants to record information about their habitual sleep over the past 4 weeks, including questions about bedtime and sleep duration.^9^ Lower overall scores are indicative of better sleep quality, with scores >5 indicative of significant sleep disturbances. Scores >5 have been proven to adequately distinguish sleep disorder patients from controls with a sensitivity of 89.6% and of specificity of 86.5%.^9^ In addition, the PSQI has been used previously in research in youth athletes.^2,30^

The Morningness-Eveningness Scale for Children (MESC) was used to determine the chronotype of the participants.^10^ This consists of 10 questions assessing their preference for mornings or evenings (eg, How alert are you in the first half hour you’re up?). Each answer is scored on a scale of 1 and 5; these are then summed to create an overall score between 10 and 42. Scores between 10 and 20 indicate an evening type, scores between 28 and 42 indicate a morning type, and scores between 21 and 27 are classified as intermediate. This scale has been validated for use in adolescents.^33^

### Daily Sleep Measures

Objective sleep measures were obtained via an Actigraph wGT3X-BT or GT9X-link accelerometer (Actigraph). These were configured with an epoch length of 60 seconds and sampling rate of 30 Hz. Each participant was required to wear the Actigraph on their nondominant wrist at night before they went to sleep and to take it off in the morning when they woke up. At the conclusion of data collection, data from each Actigraph were downloaded using the Actilife software (Actilife Version 6.13.3, Actigraph). Having been validated previously in adolescent populations, the Sadeh algorithm was used to report on sleep measures,^35^ with identified bed- and wake times verified against sleep diaries as recommended.

Subjective measures of sleep were obtained through daily self-reported sleep diaries completed at night on REDCap (Research Electronic Data Capture).^18,17^ The sleep diary asked participants to document their bedtime, wake up time, rate their subjective sleep quality on a 5-point Likert scale (1, very good; 2, good; 3, average; 4, poor; 5, very poor), and perception of pre-sleep fatigue and postawakening fatigue on a 7-point Likert scale (1, fully alert, wide awake; 7, completely exhausted). The sleep diary also asked participants to provide the number of training sessions completed each day. Sleep outcomes derived from actigraphy, and sleep diaries included:

- sleep latency (minutes) (Actigraphy): duration between diary reported bedtime and initial sleep onset;
- total sleep time (minutes) (Actigraphy): total time spent asleep from initial sleep onset to get up time;
- time in bed (minutes) (Actigraphy): total time in bed from bedtime to get up time;
- time of sleep onset (hour:minute [HH:MM]) (Actigraphy): time-of-day the participant fell asleep after going to bed;
- wake after sleep onset (minutes) (Actigraphy): time between sleep onset and get-up time spent awake;
- sleep efficiency (%) (Actigraphy): total sleep time expressed as a percentage of time in bed;
- bedtime (HH:MM) (Actigraphy): time of day the participant began attempting to sleep;
- get-up time (HH:MM) (Actigraphy): time of day the participant stopped attempting to sleep;
- subjective sleep quality (Sleep diary): quality of the previous night’s sleep on a scale of 0 to 5, with lower scores indicating better sleep quality;
- perception of pre-sleep fatigue (Sleep diary): fatigue rating before going to bed on a scale on 0 to 7, with higher scores indicating higher fatigue levels,
- postawakening fatigue (Sleep diary): fatigue rating upon awakening on a scale of 0 to 7 with higher scores indicating higher fatigue levels.

### Well-being

Measures of athlete well-being were obtained using the Multicomponent Training Distress Scale (MTDS) and the Short Recovery Stress Scale (SRSS).^22,29^ The MTDS is a 22-item questionnaire that has been validated previously in athletes.^29^ A higher score for each of the factors of depressed mood, physical symptoms, sleep disturbance, stress, fatigue, and vigor indicated a greater frequency of these feelings over the previous 24-hour period. Each question is scored on a 5-point scale (0, not at all; 1, a little; 2, moderately; 3, quite a bit; 4, extremely). The SRSS is made up of 8 items, which participants rate on a 7-point scale from 0 (does not apply at all) to 6 (fully applies).^22^ The sum of these items creates 2 subscales: stress and recovery, in which participants rate their current feelings. Higher scores in the recovery subscale indicate the athlete is feeling more recovered, whereas higher scores in the stress subscale indicate the athlete is feeling more stressed. The SRRS has been validated previously in elite sports,^24^ and has also been used in studies with youth athletes.^23,25^ These questionnaires were sent to athletes via text every Monday morning at 7:00 a.m. Participants were sent reminders if they had not completed the surveys within 24 hours. This was done through text messages sent on Tuesday morning and again on Wednesday morning if they had not yet completed it.

### Statistical Analyses

Data were analyzed using the Statistical Package for the Social Sciences (SPSS Version 25.0, IBM Corp). Data were not distributed normally as assumptions of normality for all variables were violated as measured by the Kolmogorov-Smirnov test. Separate chi-square analyses were completed that compared the proportion of male and female athletes who did not achieve >8 hours sleep and >85% sleep efficiency.^19,32^ For the MTDS, the subscale vigor was inverse coded to align with the other subscale measures (ie, a lower vigor score obtains a ‘higher’ value as it is a negative outcome). In addition, MTDS subscales as well as the total score (ie, sum of all subscales) were used in the analysis. Baseline sleep, daily sleep, and weekly well-being trends were examined through descriptive analysis using means and standard deviations. Generalized estimating equations (GEE) were used to assess the relationship between each sleep and well-being variable. Given that GEE assumes missing data are missing completely at random (MCAR), the Kruskal-Wallis test was used to evaluate whether the participant-level missing data follow the MCAR assumption. For each GEE, subject effect was participant, within-subject effect was week number, and the well-being variable included the dependent variable. The sex of the youth athletes was included as a factor, and the specific sleep variable was the covariate in the models. Analyses examined the relationship between well-being variables and (1) the previous night’s sleep, and (2) the mean of the previous 5 nights. This included the variables of total sleep time, sleep efficiency, latency, wake after sleep onset, bedtime, onset time, and wake up time. In addition to sleep rating, fatigue rating immediately before bed and fatigue rating upon awakening were measured through sleep diaries. This was done to determine whether there was a cumulative effect of sleep on well-being. Beta values were reported, indicating the change in the dependent variable for each 1-unit increase in the predictor variable. A Mann-Whitney *U* test was performed to examine differences in sleep and wellbeing variables between participants classified by the PSQI as “with significant sleep disturbances” and those classified as “without significant sleep disturbances”. For all analyses, the *P* value was set at <0.05.

## Results

Across the study, there were 3417 nights (of a possible 5488 nights) of sleep recorded from a total of 98 youth athletes. The breakdown across days of the week is as follows: Monday nights, 14% (n = 489); Tuesday nights, 15% (n = 516); Wednesday nights, 16% (n = 543); Thursday nights, 15% (n = 503); Friday nights, 15% (n = 515); Saturday nights, 12% (n = 423); Sunday nights, 13% (n = 428). Participants had a significantly longer total sleep time on Saturday night (mean, 7 hours 13 minutes; SD, 1 hour 13 minutes) compared with the other nights of the week. Participants completed an average of 4.15 sessions (training and matches) per week. The average PSQI value was 5.3 (±3), with 39 participants (40%) having values >5, indicative of significant sleep disturbances. The average ABSQ value across participants was 36 (±8 arbitrary units [au]). Most participants (n = 72) reported a morning chronotype, whereas 17 were considered intermediate and 6 were considered evening; 3 did not complete the chronotype questionnaire.

### Sleep Characteristics

Sleep outcomes are presented in Table 1. Participants averaged 7.00 hours of sleep each night and had an average sleep onset time of 11:07 p.m. Female athletes had <8 hours sleep on 82% of the nights studied compared with 86% for male athletes (*P* = 0.002). In addition, female athletes had <85% sleep efficiency on 45% of the nights studied compared with 60% for male athletes (*P* < 0.001). Sex differences were observed for fatigue rating upon awakening, fatigue rating immediately before bed, latency, efficiency, wake after sleep onset, and bedtime. Female athletes had a later bedtime, longer sleep onset latency, and had a greater wake after sleep onset. Female athletes also had a higher fatigue rating upon awakening and fatigue rating immediately before bed.

**Table 1.** Sleep variables including subjective sleep responses and objective data and intraindividual variations

	Overall	Female	Male	*P* value
Subjective sleep quality, au	2.28 (0.97)	2.41 (0.98)	2.16 (0.95)	0.50
Fatigue rating upon awakening, au	3.60 (1.43)	3.82 (1.36)	3.41 (1.46)*	0.03
Fatigue rating immediately before bed, au	4.05 (1.40)	4.34 (1.39)	3.79 (1.37)*	0.002
Total time in bed, hours*^a^*	8.38 (1.16)	8.30 (1.21)	8.44 (1.11)	0.28
Total sleep time, hours*^a^*	7.00 (1.08)	7.05 (1.13)	6.96 (1.04)	0.53
Sleep latency, minutes*^a^*	13.19 (13.30)	11.83 (12.99)	14.41 (13.46)*	0.04
Sleep efficiency, %*^a^*	83.70 (6.90)	84.94 (6.59)	82.58 (6.98)*	0.01
Wake after sleep onset, minutes*^a^*	69.10 (34.46)	63.63 (32.56)	74.00 (35.31)*	0.02
Bedtime, HH:MM*^a^*	22:53 (1.10)	23:04 (1.05)	22:44 (1.13)*	0.04
Sleep onset time, HH:MM*^a^*	23:07 (1.11)	23:16 (1.05)	22:59 (1.15)	0.07
Wake up time, HH:MM*^a^*	07:16 (1.09)	07:22 (1.10)	07:10 (1.08)	0.14

Data presented as mean (SD) unless otherwise noted. All time variables are using 24-hour clock. Comparisons were analyzed using GEEs. au, arbitrary units; GEE, generalized estimating equation.

a Measures were taken using an Actigraph.

* Statistically significant difference from female athletes (*P* < 0.05).

### Well-being Characteristics

Measures of well-being are presented in Table 2. There was no significant difference between male and female athletes with regards to overall score on the MTDS. However, male athletes reported significantly higher vigor (scores inversed) and lower perceived stress than female athletes. Male athletes also reported higher recovery scores as measured by the SRSS. There were no other significant sex differences for measures of well-being.

**Table 2.** Well-being variables from the MTDS and SRSS

	Overall	Female	Male	*P* values
MTDS				
Depression	2.23 (2.95)	2.59 (3.18)	1.95 (2.63)	0.24
Vigor*^a^*	6.49 (3.04)	7.18 (2.88)	5.80 (3.03)*	0.01
Physical symptoms	4.09 (3.07)	3.84 (3.09)	4.50 (3.02)	0.13
Sleep disturbance	1.97 (2.25)	2.05 (2.24)	1.86 (2.22)	0.57
Stress	2.98 (3.11)	3.77 (3.41)	2.15 (2.55)*	0.002
Fatigue	4.31 (2.98)	4.29 (3.04)	4.41 (2.94)	0.75
Overall	20.40 (11.61)	21.64 (12.55)	19.10 (10.39)	0.23
SRSS				
Recovery*^b^*	15.12 (4.32)	14.12 (4.20)	15.97 (4.35)*	0.01
Stress	8.53 (4.94)	9.07 (4.98)	7.81 (4.89)	0.10

Data presented as mean (SD). Lower scores indicated better well-being and stress scores, unless indicated. Comparisons were analyzed using GEEs. GEE, generalized estimating equation; MTDS, multicomponent training distress scale; SRSS, short recovery stress scale.

a Inverse value presented.

b A higher score led to better recovery.

* Statistically significant difference from female athletes (*P* < 0.05).

### Associations Between Sleep and Subjective Wellbeing

The associations between sleep the night before and well-being are shown in Table 3. Results based on mean sleep for the previous 5 nights are included in Table 4. Participant-level missing data were evaluated for the subjective sleep, objective sleep, MTDS, and SRSS variables. The analysis revealed no significant difference in missing data across different week numbers.

**Table 3.** Results of GEE of the sleep 1 night before well-being testing

	Total sleep time, hours*^a^*	Sleep efficiency, %*^a^*	Latency, minutes*^a^*	Wake after sleep onset, minutes*^a^*	Sleep rating, au*^b^*	Fatigue rating immediately before bedtime, au*^b^*	Fatigue rating upon awakening, au*^b^*	Bedtime*^b^*	Onset time*^a^*	Wakeup time*^a^*
MTDS										
Depression subscale	−0.50 (−0.95, −0.05) 0.03*	0.01 (−0.05, 0.07) 0.68	−0.01 (−0.03, 0.01) 0.32	−<0.01 (−0.02, 0.01) 0.37	0.85 (0.50, 1.19) <0.001*	0.39 (0.16, 0.63) <0.01*	0.50 (0.25, 0.74) <0.001*	0.56 (0.15, 0.97) <0.01*	0.51 (0.09, 0.94) 0.02*	0.02 (−0.46, 0.50) 0.94
Vigor subscale	−0.21 (−0.56, 0.14) 0.25	0.03 (−0.03, 0.08) 0.37	−0.01 (−0.02, 0.01) 0.44	−0.01 (−0.02, 0.01) 0.36	0.65 (0.28, 1.03) <0.001*	0.26 (−0.03, 0.54) 0.08	0.30 (0.03, 0.58) 0.03*	0.40 (0.07, 0.73) 0.02*	0.37 (0.05, 0.69) 0.03*	0.13 (−0.17, 0.42) 0.41
Physical Symptoms subscale	−0.26 (−0.61, 0.08) 0.13	−0.02 (−0.09, 0.05) 0.55	−0.01 (−0.04, 0.01) 0.38	0.01 (−0.01, 0.02) 0.45	0.60 (0.13, 1.08) 0.01*	0.57 (0.22, 0.92) <0.01*	0.55 (0.27, 0.83) <0.001*	0.21 (−0.13, 0.55) 0.23	0.17 (−0.14, 0.49) 0.28	0.03 (−0.29, 0.34) 0.88
Sleep disturbance subscale	−0.62 (−0.97, −0.27) <0.001*	−0.03 (−0.08, 0.02) 0.30	−0.01 (−0.02, 0.01) 0.36	<0.001 (−0.01, 0.01) 0.82	1.04 (0.72, 1.35) <0.001*	0.27 (0.08, 0.45) <0.01*	0.47 (0.28, 0.66) <0.001*	0.56 (0.22, 0.90) <0.01*	0.53 (0.19, 0.87) <0.01*	0.02 (−0.34, 0.38) 0.92
Stress subscale	−0.78 (−1.23, −0.33) <0.001*	<0.001 (−0.06, 0.07) 0.90	−0.02 (−0.03, <0.001) 0.09	−0.01 (−0.02, 0.01) 0.39	0.77 (0.34, 1.20) <0.001*	0.47 (0.17, 0.77) <0.01*	0.70 (0.42, 0.97) <0.001*	0.69 (0.25, 1.13) <0.01*	0.63 (0.18, 1.08) <0.01*	−0.13 (−0.68, 0.43) 0.65
Fatigue subscale	−0.29 (−0.66, 0.07) 0.11	0.01 (−0.05, 0.08) 0.71	−0.02 (−0.04, 0.01) 0.12	−<0.001 (−0.01, 0.01) 0.74	0.68 (0.23, 1.14) <0.01*	0.68 (0.38, 0.99) <0.001*	0.77 (0.50, 1.04) <0.001*	0.22 (−0.09, 0.53) 0.17	0.16 (−0.16, 0.48) 0.32	−0.13 (−0.50, 0.25) 0.51
Overall	−2.57 (−4.27, −0.87) <0.01*	<0.001 (−0.26, 0.26) 0.99	−0.06 (−0.15, 0.02) 0.12	−0.01 (−0.06, 04) 0.68	4.39 (2.79, 5.99) <0.001*	2.53 (1.39, 3.68) <0.001*	3.19 (2.21, 4.17) <0.001*	2.49 (1.04, 3.94) <0.001*	2.24 (0.70, 3.78) <0.01*	−0.10 (−2.00,1.81) 0.92
SRSS										
Recovery	0.41 (−0.13, 0.95) 0.14	0.01 (−0.07, 0.10) 0.77	−<0.001 (−0.03, 0.02) 0.73	<0.001 (−0.02, 0.02) 0.98	−1.02 (−1.64, −0.41) <0.01*	−0.20 (−0.61, 0.20) 0.33	−0.33, (−0.77, 0.11) 0.14	−0.42 (−0.86, 0.02) 0.06	−0.41 (−0.84, 0.01) 0.06	−0.08 (−0.48, 0.32) 0.68
Stress	−0.82 (−1.50, −0.15) 0.02*	−0.02 (−0.13, 0.08) 0.67	−0.02 (−0.04, 0.01) 0.18	<0.001 (−0.02, 0.02) 0.68	1.67 (0.91, 2.43) <0.001*	1.23 (0.74, 1.71) <0.001*	1.26 (0.79, 1.74) <0.001*	0.95 (0.36, 1.55) <0.01*	0.81 (0.19, 1.42) 0.01*	0.20 (−0.55, 0.95) 0.60

Data presented as β (95% CI) *P* value. au, arbitrary units; GEE, generalized estimating equation; MTDS, multicomponent training distress scale; SRSS, short recovery stress scale.

a Measures derived from actigraphy.

b Measures derived from sleep diary.

* Statistical significance of *P* < 0.05.

**Table 4.** Results of GEE of sleep over the average of 5 nights before well-being testing

	Total sleep time, hours^a^	Sleep efficiency, %*^a^*	Latency, minutes*^a^*	Wake after sleep onset, minutes^a^	Sleep rating, au*^b^*	Fatigue rating immediately before bedtime, au*^b^*	Fatigue rating upon awakening, au*^b^*	Bedtime*^a^*	Onset Time*^a^*	Wakeup time*^a^*
MTDS										
Depression subscale	−0.49 (−0.94, −0.03) 0.04*	0.03 (−0.06, 0.12) 0.54	−0.03 (−0.06, 0.01) 0.14	−0.01 (−0.03, 0.01) 0.38	1.03 (0.58, 1.49) <0.001*	0.52 (0.18, 0.86) <0.01*	0.63 (0.31, 0.95) <0.001*	0.57 (0.10, 1.00) 0.02*	0.53 (0.06, 1.00) 0.03*	0.13 (−0.42, 0.68) 0.64
Vigor subscale	0.04 (−0.51, 0.59) 0.89	0.08 (<0.01, 0.16) 0.05*	−0.01 (−0.05, 0.03) 0.53	−0.02 (−0.03, −<0.01) 0.02*	0.71 (0.17, 1.24) 0.01*	0.54 (0.16, 0.91) 0.01*	0.51 (0.13, 0.90) 0.01*	0.16 (−0.21, 0.52) 0.40	0.14 (−0.22, 0.49) 0.45	−0.08 (−0.54, 0.39) 0.75
Physical Symptoms subscale	−0.41 (−0.96, 0.15) 0.16	−0.04 (−0.14, 0.07) 0.51	−0.01 (−0.05, 0.03) 0.61	0.01 (−0.02, 0.03) 0.59	0.45 (−0.19, 1.08) 0.17	0.61 (0.19, 1.04) <0.01*	0.71 (0.35, 1.08) <0.001*	0.43 (−0.05, 0.91) 0.08	0.41 (−0.06, 0.88) 0.09	0.28 (−0.19, 0.76) 0.24
Sleep disturbance subscale	−0.61 (−1.04, −0.17) 0.01*	−0.04 (−0.11, 0.03) 0.23	0.01 (−0.03, 0.04) 0.78	<0.01(−0.01, 0.01) 0.65	10.07 (0.71, 1.43) <0.001*	0.37 (0.12, 0.62) <0.01*	0.53 (0.28, 0.77) <0.001*	0.49 (04, 0.94) 0.04*	0.49 (0.04, 0.94) 0.03*	0.14 (−0.37, 0.65) 0.59
Stress Subscale	−0.76 (−1.31, −0.20) 0.01*	0.01 (−0.08, 10) 0.83	−0.01 (−0.06, 0.03) 0.57	−0.01 (−0.03, 0.01) 0.33	0.97 (0.46, 1.49) <0.001*	0.63 (0.20, 1.05) <0.01*	0.87 (0.51, 1.22) <0.001*	0.68 (0.11, 1.25) 0.02*	0.66 (0.09, 1.23) 0.02*	0.03 (−0.64, 0.71) 0.92
Fatigue subscale	−0.53 (−1.01, −0.04) 0.04*	0.01 (−0.10, 0.11) 0.93	−0.02 (−0.07, 0.02) 0.37	−<0.01 (−0.03, 0.02) 0.73	0.65 (0.07, 1.23) 0.03*	0.87 (0.47, 1.27) <0.001*	0.98 (0.65, 1.30) <0.001*	0.27 (−0.19, 0.73) 0.26	0.24 (−0.27, 0.70) 0.31	−0.22 (−0.78, 0.34) 0.43
Overall	−2.69 (−4.67, −0.79) 0.01*	0.03 (−0.36, 0.42) 0.88	−0.07 (−0.24, 0.10) 0.40	−0.03 (−0.10, 0.05) 0.53	4.69 (2.57, 6.77) <0.001*	30.35 (1.88, 4.83) <0.001*	4.08 (2.80, 5.35) <0.001*	2.51 (0.40, 4.61) 0.02*	2.38 (0.27, 4.48) 0.02*	0.31 (−2.11, 2.72) 0.80
SRSS										
Recovery	0.45 (−0.40, 1.30) 0.30	0.04 (−0.08, 0.16) 0.52	−0.01 (−0.09, 0.06) 0.72	−0.01 (−0.03, 0.02) 0.61	−1.06 (−1.96, −0.16) 0.02*	−0.32 (−0.94, 0.30) 0.31	−0.45 (−1.10, 0.20) 0.17	−0.23 (−0.75, 0.29) 0.38	−0.25 (−0.76, 0.27) 0.35	−0.03 (−0.61, 0.55) 0.92
Stress	−0.87 (−1.90, 0.16) 0.10	−0.06 (−0.24, 0.13) 0.56	−0.03 (−0.11, 0.05) 0.44	0.01 (−0.03, 0.04) 0.64	1.95 (0.82, 3.07) <0.001*	1.62 (0.98, 2.24) <0.001*	1.57 (0.94, 2.21) <0.001*	1.03 (0.14, 1.93) 0.02*	0.98 (0.08, 1.89) 0.03*	0.71 (−0.32, 1.74) 0.18

Data presented as β (95% CI) *P* value. au, arbitrary units; GEE, generalized estimating equation; MTDS, multicomponent training distress scale; SRSS, short recovery stress scale.

a Measures derived from actigraphy.

b Measures derived from sleep diary.

* Statistical significance of *P* < 0.05.

#### Previous Night Sleep and Subjective Wellbeing

Total sleep time was associated negatively with some MTDS scores (ie, indicating compromised well-being), with associations found for the depression, stress, and sleep disturbance subscales and overall total score. However, no associations were found between total sleep time and the vigor, physical symptoms, and fatigue subscales. Total sleep time was also associated negatively with the SRSS stress scale (ie, indicating greater stress), but not the recovery subscale. Subjective sleep ratings were associated with MTDS scores, with all 6 MTDS subscales and the total MTDS score associated positively with subjective sleep quality. For the SRSS, subjective sleep quality was associated positively with the stress subscale (ie, indicating greater stress) and negatively with the recovery scale. Fatigue ratings immediately before bedtime were associated positively with MTDS scores (ie, poorer well-being), with all 6 subscales and total score having statistically significant associations. For the SRSS, fatigue ratings immediately before bedtime were associated positively with the stress subscale, but not the recovery subscale. Fatigue rating upon awakening (higher rating indicating higher fatigue levels) was also associated positively with MTDS scores, with all 6 subscales and total score significantly associated. For the SRSS, fatigue ratings upon awakening were associated positively with the stress subscale, but not the recovery subscale. Bedtime and sleep onset time were associated with increases in MTDS scores including the subscales of depression, vigor (scores inversed), sleep disturbance, stress, and the overall score. However, there were no associations between bedtime and sleep onset times and the subscales of physical symptoms and fatigue. For the SRSS, bedtime and sleep onset time were associated positively with the stress subscale. No associations were found between sleep efficiency, latency, wake after sleep onset, wake-up time, and any well-being measures.

### Previous 5 Nights and Subjective Wellbeing

Total sleep time was associated negatively with MTDS scores, with associations found for the depression, sleep disturbance, stress, fatigue subscales, and overall total score. However, no associations were found between total sleep time and the vigor or physical symptoms subscales of the MTDS or any of the SRSS subscales. Increases in sleep efficiency were associated positively with the vigor subscale and increases in wake after sleep onset were associated negatively with the vigor subscale, but no other associations were present for efficiency and WASO. These findings are different to the associations with well-being testing for sleep the 1 night before, where no associations were observed between efficiency or WASO and any well-being measures. Subjective sleep ratings were associated with higher MTDS scores, with associations found for the depression, vigor, sleep disturbance, stress, and fatigue subscales and overall total score, but not the physical symptoms subscale. For the SRSS, subjective sleep quality was associated positively with the stress subscale (ie, indicating greater stress) and negatively with the recovery scale. Fatigue ratings immediately before bedtime were associated positively with MTDS scores (ie, poorer well-being), with all 6 subscales and total score having associations. For the SRSS, fatigue ratings immediately before bedtime were associated positively with the stress subscale, but not the recovery subscale. Fatigue rating upon awakening (higher rating indicating and higher fatigue levels) was also associated positively with MTDS scores, with all 6 subscales and total score significantly associated. For the SRSS, fatigue ratings upon awakening were positively associated with the stress subscale, but not the recovery subscale. Bedtime and sleep onset time were associated positively with MTDS scores, including the subscales of depression, sleep disturbance, and stress and overall total score. However, bedtime and sleep onset time were not associated with the vigor, physical symptoms, or fatigue subscales. For the SRSS, bedtime and sleep onset time were associated positively with the stress subscale, but not the recovery subscale. There were no associations between latency or wake-up time and any well-being measures.

### Well-being in Participants With and Without PSQI-Defined Sleep Problems

As shown in Table 5, participants that were classified as with “significant sleep disturbances” on the PSQI score significantly higher on all measures of the MTDS (indicating poorer wellbeing) when compared with those without. In addition, the recovery subscale of the SRSS was significantly lower, and the stress subscale was significantly higher in youth athletes with “significant sleep disturbances.”

**Table 5.** Well-being variables from the MTDS and SRSS in participants with, and without, PSQI-defined sleep problems

	With significant sleep disturbances	Without significant sleep disturbances	*P* values
MTDS			
Depression	3.40 (3.61)	1.43 (2.06)*	0.01
Vigor*^a^*	6.91 (2.53)	6.14 (3.29)*	<0.01
Physical Symptoms	4.71 (3.08)	3.86 (3.02)*	<0.01
Sleep disturbance	2.81 (2.51)	1.40 (1.83)*	<0.01
Stress	4.74 (3.67)	1.72 (1.86)*	<0.01
Fatigue	5.22 (3.13)	3.80 (2.76)*	<0.01
Overall	26.16 (13.04)	16.73 (8.66)*	<0.01
SRSS			
Recovery*^b^*	13.74 (3.62)	15.99 (4.59)*	<0.01
Stress	10.60 (4.61)	6.98 (4.68)*	<0.01

Data presented as mean (SD). Lower scores indicated better wellbeing and stress scores, unless otherwise indicated. Comparisons were analyzed using a Mann-Whitney U test. MTDS, multicomponent training distress scale; PSQI, Pittsburgh sleep quality index; SRSS, short recovery stress scale.

a Inverse value presented.

b Higher score led to better recovery.

* Statistically significantly different from significant sleep disturbances group.

## Discussion

The aim of this study was to characterize sleep and well-being in youth athletes and examine the relationships between measures of sleep and subjective well-being. In over 80% of the nights studied, youth athletes were not achieving the recommended 8 to 10 hours of sleep per night. Many youth athletes were identified as having significant sleep disturbances. Less sleep, as measured by actigraphy, and poorer subjective sleep quality were associated with greater subjective stress and overall poorer well-being in youth athletes.

This study highlighted that the sleep of youth athletes was suboptimal, with 40% of youth athletes identified as having significant sleep disturbances. This is being echoed in adolescents around the world.^15,40,52^ Furthermore, total sleep time in this study was lower than the recommended 8 to 10 hours.^19^ Participants failed to achieve 8 hours of sleep on >80% of the nights studied. This study captured over 3400 nights, evenly spread across all days/nights of the week. However, similar findings have been identified across previous research with youth athletes consistently obtaining, on average, <8 hours sleep.^2,26,42^ Comparable research monitoring a large number of youth athletes (n = 128) collecting approximately 896 nights of data identified that youth athletes were obtaining, on average, 7.1 hours of sleep; however, this was only across a period of around 7 days.^2^ In nonathlete adolescent populations, Short et al^39^ noted that participants achieved an average of 8 hours and 17 minutes sleep on weekdays, and 8 hours and 40 minutes of sleep on weekends. These differences in total sleep time could be attributed to the reduced opportunities for sleep in youth athletes, both during the week and on weekends due to their sport commitments in addition to school, part-time work, and social commitments.

An increase in total sleep time the night before assessing well-being showed improvements in multiple aspects of well-being as measured by the MTDS (ie, depressed moods, perceived sleep disturbances, and perceived stress subscales). This is consistent with previous research in youth athletes that noted improvements in well-being with better sleep behaviors.^37,46^ The current study also identified that an increase in sleep time was associated with a decrease in perceptions of stress as measured by the SRSS. This aligns with previous research by Kölling et al,^25^ who reported youth rowers with an extended night of total sleep time showed improved stress and recovery scores on the SRSS. Their results differ to the current study where sleep was not associated with next-day recovery status. Rowers in the study by Kölling et al^25^ were likely training at extremely high levels in preparation for the World Championships; thus discrepancies in training intensity between these elite rowers, and the subelite athletes examined in the present study may explain the different findings. For example, sleep may become more important for perceived recovery as training intensities increase.

It is not just the total time of sleep that youth athletes achieve, it is also the timing of that sleep that is important. In the current study, a later bedtime and sleep onset time resulted in poorer well-being scores as measured by the MTDS. The average bedtime of youth athletes in the current study was 10:53 p.m. This is similar to other research where a cohort of youth track and field athletes had a bedtime of 10:55 p.m.^34^ Other studies with youth athletes from the sports of archery, golf, swimming, cycling, badminton, fencing, soccer, handball, and volleyball reported bedtimes ranging from 11:06 p.m. to 12:42 a.m.^2^ These bedtimes could be due to the times of their training and games,^7^ and highlights the pervasive nature of this challenge. Future research could explore the impact of the timing of trainings and games on sleep and subsequent well-being in youth athletes. Noting the compromised well-being associated with later bedtimes in the current study, this suggests that the later bedtimes in youth athletes could be negatively impacting their well-being. Considering youth may be at risk of poorer well-being for a host of reasons,^51^ it is important to identify any factors that may be compromising their well-being and prioritize mitigation strategies that may be able to improve overall well-being, such as education on the importance of earlier bedtimes. Research has identified that youth athletes went to bed significantly later after evening training compared with morning training.^7^ Bedtimes could also be impacted by using electronic devices before bed and social media use.^1^ Where possible, parents and coaches should encourage earlier bedtimes to maximize opportunities for sleep, given the benefits to well-being for these athletes. In addition, to facilitate longer total sleep times, later school start times could be encouraged. Considering the impact of training schedules and electronic devices, future research should investigate the relationship between sleep, training schedules, electronic devices, and the well-being of youth athletes. Future research could also seek to examine the differences in these results between sports.

Similar to previous research in other contexts,^8^ it was often the perception of fatigue and sleep quality that had the greatest impact on measures of well-being. Specifically, there were a greater number of associations between subjective measures of sleep and perceived well-being than the objective measures of sleep and perceived well-being. Watson and Brickson^46^ identified that self-reported measures of mood, soreness, and stress all improved significantly after nights with greater subjective sleep quality and sleep duration. This relationship between subjective measures of well-being and subjective measures of sleep may indicate that perceived sleep is more sensitive in detecting changes in subjective well-being compared with objective sleep measures. Previous research has established that adolescents at higher risk for clinical depression and anxiety often report poor sleep quality.^15^ Therefore, subjective sleep questionnaires may be a low cost and easy to administer solution for coaches and sport administrators to use to monitor their youth athletes’ sleep, from which some inferences of their well-being may be considered. This is important for sporting clubs that may not have the money or resources to invest in wearable devices to objectively track sleep and would give coaches direct information regarding their sleep.

Differences were observed between sexes for well-being measures with female youth athletes likely to be more stressed, and report lower energy and poorer recovery when compared with male athletes. Previous research has suggested that female athletes will report symptoms more frequently and are more likely to perceive additional difficulties than male athletes when exposed to the same stressors.^21^ This is consistent with the current study where higher perceived stress scores were reported among female athletes. In addition, previous research in the general population has identified that female athletes are more likely to develop anxiety than male athletes,^28^ and this could explain the differences in well-being observed in this study. Potential mechanisms for these differences have been associated with the differing hormone levels between the sexes.^28^ In addition, the disparity could also be due to girls exhibiting different control strategies and metacognitive beliefs.^5^ Consistent with previous research,^2,41,43^ male athletes in the current study reported lower pre- and post-bed perceived fatigue levels, earlier bedtimes, and longer total sleep times. In comparison, female athletes reported shorter sleep onset latency, lower wake after sleep onset, and higher sleep efficiency, suggesting that, although they may not sleep as long as their male counterparts, they are achieving better efficiency, including waking less throughout the night and falling asleep quicker.

### Limitations

A major limitation of this study was participant compliance and missing data from the participants included in the analysis (37.7% objective data missing; 22.4% subjective sleep and fatigue ratings missing; 11% well-being data missing). However, to minimize the effect of missing data, participants had to complete 50% to be included in the analysis. This study was designed to be completed remotely, which relied on participants being proactive in wearing their actigraph to measure sleep every night and adhering to the scheduled questionnaires. To try and prevent missing datapoints, participants were sent the daily sleep questionnaire late in the evening when they were likely to be preparing for bed. They were also sent reminders if they had not completed the well-being questionnaires within 24 hours. In addition, due to the large number of youth athletes involved in this study, it was not feasible to collect all data at once. As a result, the time of year likely impacted youth athletes’ sleep and well-being—a factor that was not controlled for.

## Conclusion

Youth athletes from across a range of various sports did not meet the recommended sleep duration for their age, and many were above the threshold for established “sleep problems.” An increase in total sleep time resulted in improved subjective well-being and a reduction in perceived stress. Conversely, a later bedtime, poorer perceived sleep quality, and higher fatigue levels were associated with reduced well-being. Female athletes tended to report poorer recovery, higher stress levels, and lower energy than male athletes. Educating youth athletes, their families, and coaches on the importance of adequate sleep and sleep hygiene practices is needed. This could include emphasizing the value of getting to bed at a reasonable time to ensure it does not detrimentally impact their mental health and well-being.
